# Subpolar marginal seas fuel the North Pacific through the intermediate water at the termination of the global ocean circulation

**DOI:** 10.1073/pnas.2000658117

**Published:** 2020-05-27

**Authors:** Jun Nishioka, Hajime Obata, Hiroshi Ogawa, Kazuya Ono, Youhei Yamashita, Keunjong Lee, Shigenobu Takeda, Ichiro Yasuda

**Affiliations:** ^a^Pan-Okhotsk Research Center, Institute of Low Temperature Science, Hokkaido University, Sapporo 060-0819, Japan;; ^b^Arctic Research Center, Hokkaido University, Sapporo 001-0021, Japan;; ^c^Atmosphere and Ocean Research Institute, The University of Tokyo, Kashiwa 277-8564, Japan;; ^d^Faculty of Environmental Earth Science, Hokkaido University, Sapporo 060-0810, Japan;; ^e^Graduate School of Fisheries and Environmental Sciences, Nagasaki University, Nagasaki 852-8521, Japan

**Keywords:** dissolved iron, macronutrients, North Pacific Ocean, island chains mixing, GEOTRACES

## Abstract

The mechanism by which nutrients in the deep ocean are uplifted to maintain nutrient-rich surface waters in the subarctic Pacific has not been properly described. The iron (Fe) supply processes that control biological production in the nutrient-rich waters are also still under debate. Here, we report the processes that determine the chemical properties of intermediate water and the uplift of Fe and nutrients to the main thermocline, which eventually maintains surface biological productivity. Extremely nutrient-rich water is pooled in intermediate water (26.8 to 27.6 σ_θ_) in the western subarctic area, especially in the Bering Sea basin. Increases of two to four orders in the upward turbulent fluxes of nutrients were observed around the marginal sea island chains, indicating that nutrients are uplifted to the surface and are returned to the subarctic intermediate nutrient pool as sinking particles through the biological production and microbial degradation of organic substances. This nutrient circulation coupled with the dissolved Fe in upper-intermediate water (26.6 to 27.0 σ_θ_) derived from the Okhotsk Sea evidently constructs an area that has one of the largest biological CO_2_ drawdowns in the world ocean. These results highlight the pivotal roles of the marginal seas and the formation of intermediate water at the end of the ocean conveyor belt.

## Significance

A correct understanding of the iron and macronutrient dynamics at the termination of the global ocean conveyor belt circulation is critical for understanding the global carbon cycle and its changes in geological timescale. Newly obtained and compiled datasets of iron and macronutrients with the vertical mixing magnitude in the subarctic Pacific and marginal seas indicate the processes that determine the nutritional status of intermediate waters and the mechanisms by which subpolar marginal seas fuel the North Pacific Ocean through the intermediate water. The intermediate water formation processes play a major role in the connection of nutrients between the deep water and the surface water above it, and sustain biological production, at the termination of the global nutrient circulation.

Although the subarctic Pacific is a high-nutrient low-chlorophyll (HNLC) region, where high concentrations of macronutrients (hereafter “nutrients”) remain in the surface and phytoplankton growth is limited by iron (Fe) availability (1–3), this area has the largest biological CO_2_ drawdown among the world oceans (4), and the high productivity of the region’s ecosystem and fisheries (5) must be sustained by supplies of both Fe and nutrients into the euphotic zone.

Since the sinking of biogenic particles exports nutrients toward the intermediate/deep sea, the maintenance of surface nutrients requires a return path of the nutrients from the deep ocean (6). In the Southern Ocean, the main nutrient return path from deep water by upwelling and subsequent entrainment into sub-Antarctic mode water has been well explained (6). In the North Pacific high-latitude region, nutrients accumulate in deep water with old ^14^C age (7–9). In previous ^14^C observations in the North Pacific, the oldest water was clearly observed at ∼2,000- to 2,500-m depth, and the deep water returned southward below the intermediate water (7, 10), which had the highest nitrate and phosphate concentrations, indicating that the high-nutrient deep water does not directly affect the surface layer in the subarctic Pacific. Although previous studies imply that the nutrient return path to the surface exists in the northwest corner of the Pacific (6, 11), detailed mechanisms by which nutrients return to the surface layer and how HNLC water is formed in the North Pacific have not been described.

In addition to winter entrainment mixing, an important factor in understanding the return of nutrients to surface water is vertical turbulent diapycnal mixing. Because density stratification in the ocean generally prevents vertical transport (12), it is difficult for dense nutrient-rich deep water and shallow less-dense nutrient-depleted water to be exchanged. Therefore, vertical turbulent mixing is crucial for the quantitative evaluation of the return of nutrients from the deep layer to the surface. An important factor for controlling biological production in the nutrient-rich region is the formation of chemical properties of intermediate water (6), including nutrients and the limiting micronutrient “Fe.” North Pacific Intermediate Water (NPIW) is formed under the strong influence of the marginal seas (13–15) and may play a major role in the connection of nutrients between the deep water and the surface water above it (6). Furthermore, additional to atmospheric-dust deposition, recent trace metal measurements have highlighted the importance of localized sources of external Fe, such as river discharge, shelf sediment load, hydrothermal input, and sea ice melting (16–21). In the North Pacific, loading Fe from the continental margin and shelves of the marginal seas, from which Fe is transported by intermediate water circulations, are highlighted in recent studies (11, 22–26). There are still debates about the quantitative contributions of atmospheric dust Fe and oceanic Fe transport processes to Fe supply processes in the North Pacific (19, 27–29). To quantitatively elucidate the supply processes of Fe and nutrients to the surface in the North Pacific, it is necessary to comprehensively understand formation of the chemical properties of basin-scale intermediate water, as well as the mixing and circulation in this area (20).

**Fig. 1.**
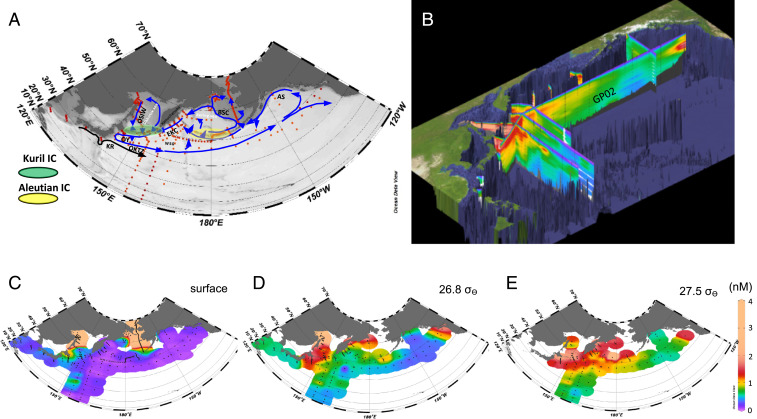
Comprehensive observation for investigating dFe in the North Pacific conducted from 1998 to 2018. (*A*) Observed stations for dataset and water current in the subarctic Pacific. (*B*) Three-dimensional dFe diagram in the North Pacific constructed by the dataset (part of the data is not included); GP02 in *B* is line ID for the GEOTRACES program. (*C*) Horizontal distribution of dFe at surface (5 to 10 m). (*D*) Same as *C*, but at isopycnal surface 26.8 σ_θ_. (*E*) Same as *C*, but at isopycnal surface 27.5 σ_θ_.

In this study, we compiled comprehensive observed data of chemical water properties (*SI Appendix*, Table S1), which includes newly obtained high-quality data from GP02 of GEOTRACES section line, the western Bering Sea, the Aleutian island chains (ICs), and the East Kamchatka Current (EKC), including dissolved Fe (dFe) and nutrients, with physical parameters of vertical mixing, in the North Pacific including the marginal seas and areas around the Kuril and the Aleutian ICs (Fig. 1*A* and *Methods*). This dataset can be used to analyze the distribution of the chemical parameters of isopycnal surfaces, and we succeed in showing the overall spatial distribution and circulation of Fe and nutrients in the North Pacific.

## Spread of Fe from the Okhotsk Sea via Ventilation

We first constructed a diagram showing the three-dimensional (3D) distribution of dFe in the North Pacific, including its subpolar marginal seas (the Okhotsk Sea and the Bering Sea) (Fig. 1*B*). From this dataset, we inferred the characteristics of dFe circulation in the North Pacific. The dFe concentration in surface waters is low throughout the subarctic Pacific region, except for in the shelf areas of the Okhotsk Sea and the Bering Sea (Fig. 1*C*). The vertical section profile of dFe along GP02 (in Fig. 1*B* and *SI Appendix*, Fig. S1*F*) was updated to cover the full section from the western to the eastern subarctic Pacific in this study. The eastern side of the subarctic Pacific has a continental shelf source of dFe along the Alaskan Stream (AS) (Fig. 1*B* and *SI Appendix*, Fig. S1*F*), as previously reported (22). This high-dFe water of the AS is basically confined to the nearshore area, because the boundary current (AS) passes along the coast, although eddy transports of the high-dFe water to offshore occasionally occur (30). The sections also clearly indicate that dFe concentrations are highest in the intermediate water on the western side of the subarctic Pacific (Fig. 1*B* and *SI Appendix*, Fig. S1*F*) as previous studies suggested (11, 24, 25).

**Fig. 2.**
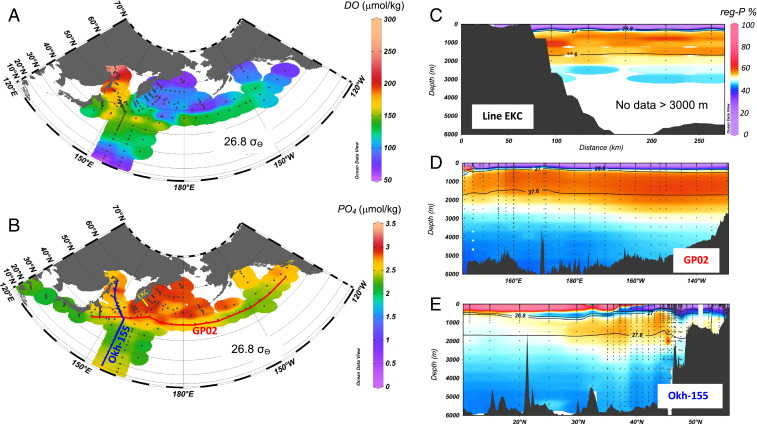
(*A*) Horizontal distribution of DO at isopycnal surface 26.8 σ_θ_. (*B*) Same as *A*, but phosphate. (*C*) Vertical section profile of proportion of regenerated phosphate along Line East Kamuchatka Current (EKC) in *B*. (*D*) Same as *C*, but along GP02 line in *B*. (*E*) Same as *C*, but along Okh-155 in *B*. [※ Nutrient includes the data referred from JAMSTEC, MR04-04 cruise data (31).] The black solid lines in *C*–*E* indicate isopycnal surfaces of 26.8, 27.0, and 27.5 σ_θ_, respectively.

The horizontal distribution indicated by isopycnal analysis in this study clearly shows evidence that the high dFe source in the intermediate waters in the western subarctic Pacific is the marginal seas. The upper (U-) NPIW density range (26.6 to 27.0 σ_θ_, where 26.8 σ_θ_ is the median density of U-NPIW) is strongly influenced by the Okhotsk Sea Intermediate Water (OSIW), whereas the lower (L-) NPIW density range (27.0 to 27.5 σ_θ_) is influenced mainly by the EKC and the Western Subarctic Gyre (WSG) (13). The isopycnal analysis clearly indicates that the dFe-rich water in the U-NPIW density range (Fig. 1*D*), in which dissolved oxygen (DO) is also higher than surrounding water (Fig. 2*A*), is derived from the OSIW that originates in the Okhotsk Sea shelf and propagates along the 26.8 σ_θ_ isopycnal surface to the western North Pacific (mainly west of 155°E) (Fig. 1*D*). In contrast, in the L-NPIW density range, e.g., at 27.5 σ_θ_ (Fig. 1*E*), dFe is high across a wide area in the western subarctic Pacific, particularly along the northern part of the WSG including the areas southeast of the Kamchatka Peninsula, the western Bering Sea basin, and around the eastern Aleutian Islands (hereafter, we define the region as “the northern WSG”) (Fig. 1*E*). The dFe distribution in the Oyashio region can be explained by the direct influence of both waters transported from the Okhotsk Sea to U-intermediate water and the EKC influence on L-intermediate water (*SI Appendix*, Fig. S2 *A*–*F*).

## Formation of Subarctic Intermediate Water Nutrient Pool

Intermediate water, which is extremely rich in phosphate (PO_4_) but low in DO, was observed on the 26.8 σ_θ_ isopycnal surface in the northern WSG (Fig. 2 *A* and *B*), especially in the western Bering Sea basin, in the southeast of the Kamchatka Peninsula (Fig. 2 *A* and *B* and *SI Appendix*, Fig. S1 *A* and *B*) and around the eastern Aleutian Islands (Fig. 2 *A* and *B*). In fact, the water was observed in the wide density range of 26.6 to 27.6 σ_θ_ (which covers both density ranges of U- and L-intermediate water) along GP02 in the entire subarctic area (*SI Appendix*, Fig. S1 *D* and *E*). In addition, the calculated percentage of regenerated (reg-) PO_4_ out of the total PO_4_ [(AOU × R_P:DO_)/observed PO_4_ × 100] (see *Methods*) in a section along the EKC line indicates that more than one-half the total PO_4_ in the density range of 26.8 to 27.6 σ_θ_ is reg-PO_4_ (Fig. 2*C*). The intermediate water with high proportion of the reg-PO_4_ is also observed in the same density range in the subarctic west-to-east section along the GP02 line (Fig. 2*D*), indicating that the reg-PO_4_–rich intermediate water is widely propagated not only in the northern WSG but also eastward to the Alaskan Gyre (Fig. 2*D* and *SI Appendix*, Fig. S1 *D* and *E*). That is, high nutrients are pooled in the subarctic intermediate water [26.8 to 27.6 σ_θ_; this is greater depth than previous definition of NPIW density range, 27.5 σ_θ_ (13)] in the northern WSG and Alaskan Gyre; we henceforth call the water the “subarctic intermediate nutrient pool” (SINP). Above the SINP, surface productive areas were observed by satellite chlorophyll images in the margin of the northern WSG along the regions of the Oyashio, southeast of the Kamchatka Peninsula, around the Kuril and the Aleutian ICs and the Bering Sea shelf slope (*SI Appendix*, Fig. S3). The formation of the chemical properties of the SINP can only be explained by the consumption of DO and regeneration of PO_4_, as particulate organic matter that sinks from the surface productive areas decomposes during the intermediate water circulation in the subarctic Pacific and its marginal seas. In contrast, the SINP formation cannot be explained by the direct transport of the nutrient-rich deep water because the deep water has a higher DO concentration.

In the U-NPIW density range, where the influence of Okhotsk Sea water is strong, a meridional vertical cross-section of the low percentage of reg-PO_4_ along Okh-155 in the Okhotsk Sea to along 155°E in the North Pacific (Fig. 2*E*) clearly indicates that newly formed (ventilated) water in the Okhotsk Sea, which has relatively low PO_4_ and high DO (Fig. 2 *A* and *B*), is distributed in the U-NPIW density range, and the U-NPIW circulation mainly transports the preformed (pre) PO_4_ onto the SINP.

## Main Nutrient Return Path from the Intermediate to the Surface

**Fig. 3.**
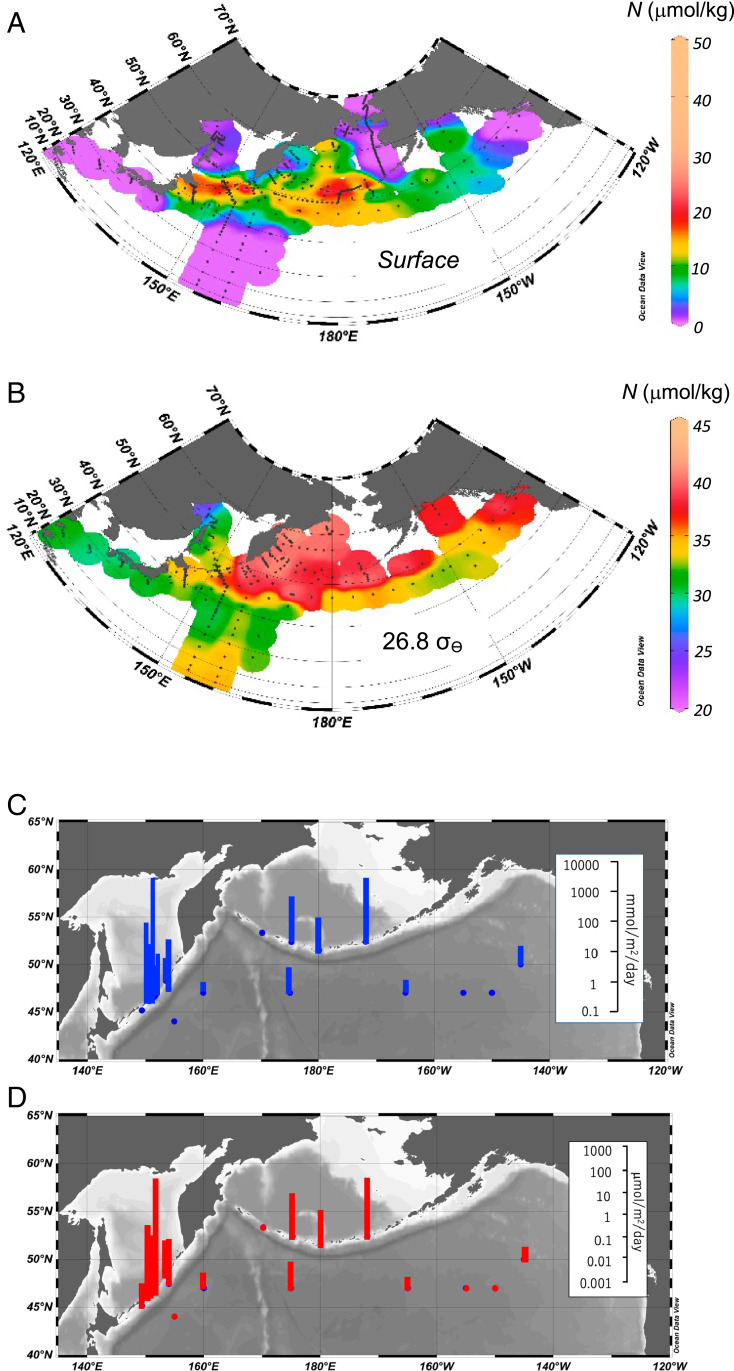
(*A*) Horizontal distribution of nitrate-plus-nitrite (N) concentration at surface (5 to 10 m). (*B*) Same as *A*, but isopycnal surface 26.8 σ_θ_. Note that the color scale is different between A and B. (*C*) Vertical upward fluxes of N around the Kuril Islands chain, Aleutian Islands chain, and the subarctic Pacific. (*D*) Same as *C*, but for dFe. [※ Nutrient for *A* and *B* includes the data referred from JAMSTEC, MR04-04 cruise data (31).]

Our dataset is mostly collected in summer season. In the dataset, the horizontal distribution of nitrate-plus-nitrite (N) concentrations near the surface (Fig. 3*A*) are variable, with concentration maxima observed around the Kuril and the Aleutian ICs, whereas the N concentrations at depth on the 26.8 σ_θ_ surface (Fig. 3*B*) (in the SINP waters) at the northern WSG are uniformly high. The surface water maxima (Fig. 3*A*) suggest that upwelling occurs around the ICs. Near these ICs, vertical turbulent fluxes of N from intermediate to surface waters, determined by direct measurements of turbulent vertical diffusivity (using average 100 to 500 m) (see *Methods*), are two to four orders of magnitude greater than those in the open ocean (Fig. 3*C* and *SI Appendix*, Table S2). The fluxes are largest in the Kuril Straits (average daily N flux, ∼100 mmol⋅m^−2^⋅d^−1^) and second largest in the Aleutian passes (average daily N flux, ∼10 mmol⋅m^−2^⋅d^−1^), and in both these regions, they are much greater than the fluxes in the subarctic Pacific (average daily N flux, ∼1 mmol⋅m^−2^⋅d^−1^) (Fig. 3*C* and *SI Appendix*, Table S2). These results indicate that the Kuril and Aleutian ICs are the hot spots that return nutrients from the intermediate water to the surface water through the enhanced turbulent diapycnal mixing caused by interactions of tidal currents with the rough topography (32–34). Accounting for the IC areas where turbulent mixing occurs, the estimated uplifted annual N fluxes around the Aleutian and the Kuril ICs [10^11^ to ∼10^13^ mol⋅y^−1^; geometric mean, ∼10^12^ mol⋅y^−1^ (*SI Appendix*, Fig. S8)] can account for less than 10% of N pooled in the SINP (4.2 ± 0.4 × 10^14^ mol) per year and comparable to the exported N from surface to below the winter mixed layer in the whole northern subarctic Pacific (∼10^12^ mol⋅y^−1^) (35, 36) (*SI Appendix*, Fig. S4), whereas the estimated flux of uplifted N only by the turbulent mixing in the open ocean in the subarctic Pacific (∼10^11^ mol⋅y^−1^; the values estimated by data obtained from KNOT, CL2-CL16; see *SI Appendix*, Fig. S7) is one order of magnitude smaller than the amount of exported N in this region. The geometric mean of total uplifted annual N flux estimated in this study is ∼10^12^ mol⋅y^−1^ (*SI Appendix*, Fig. S8). The value, however, might be underestimated or there is another missing upward flux of N (or exported N might be overestimated) because the uplifted N flux must be greater than the exported N for maintaining high nutrient surface water in the subarctic Pacific. Together with previously reported information (37), approximately <1% of N in the SINP is annually transported to NPIW. Additionally, considering the nutrient data with dFe dataset analysis, the chemical properties of uplifted intermediate waters around the Aleutian ICs have a lower dFe:N ratio than the diatom demand (see next section). These results indicate that this enhanced mixing around the Aleutian ICs, combined with winter surface mixing, plays an important role in the supply of nutrient-rich (but biologically Fe-limited) waters from the SINP to the surface and in maintaining HNLC waters in the surface layer of the subarctic Pacific and the western Bering Sea basin (see next section).

**Fig. 4.**
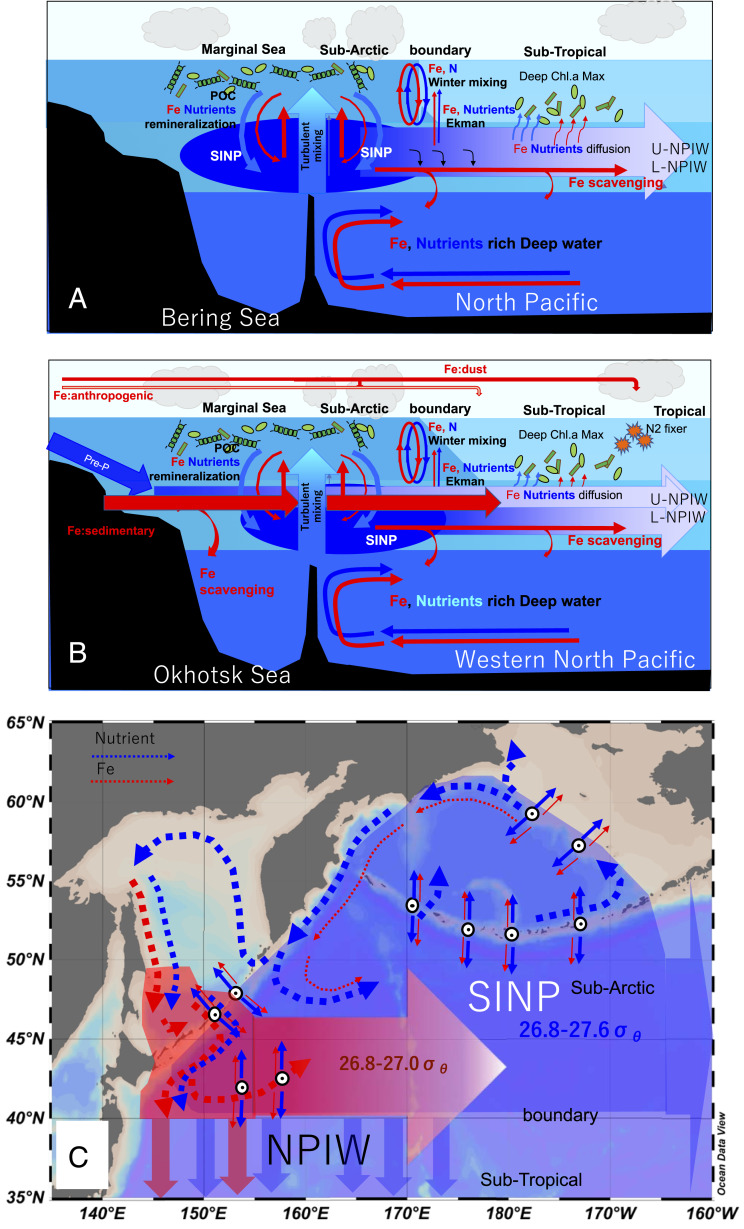
The schematic draw of the circulation and intermediate water formation processes of nutrients and dFe in the North Pacific: (*A*) through the Bering Sea, (*B*) through the Okhotsk Sea, and (*C*) horizontal circulation. Regenerated nutrients and dFe in the intermediate water are vertically supplied to surface layer by turbulent mixing around the ICs and cycle between the intermediate and the surface layer. This nutrient circulation in the intermediate water is coupled with intermediate dFe discharge from the Okhotsk Sea. Then, nutrient and Fe are transported to eastward and to low latitude by the NPIW, which influence to biological production at some hot spot in the North Pacific.

There must be another important role of mixing around the ICs. To balance the nutrient budget in the SINP, the interaction between the deep water and the intermediate water is necessary. Nutrients need to be supplied from the deep water to the SINP by the turbulent mixing processes around the ICs (Fig. 4 *A* and *B*) by the amounts that are laterally transported by the NPIW to low latitudes from the SINP (Fig. 4*C* and *SI Appendix*, Fig. S5). Understanding the nutrient transport interactions between the deep water and the intermediate water will be an issue for the future, and it is necessary to measure turbulence in the abyssal zone.

## Intermediate Water Controls Biological Productivity

**Fig. 5.**
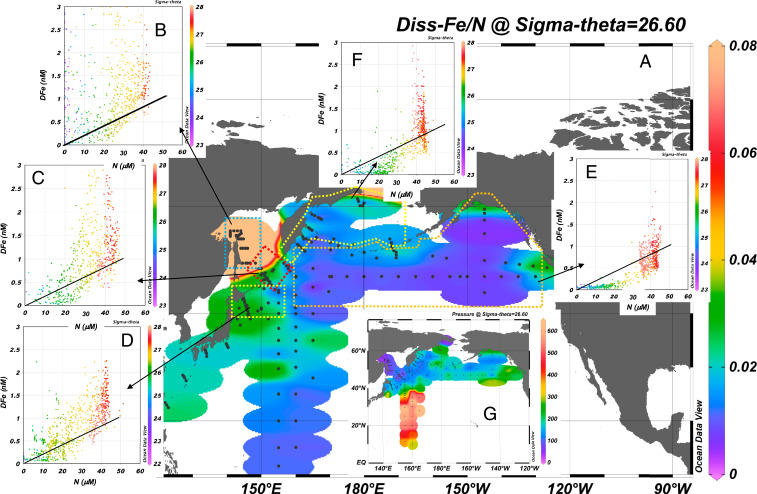
(*A*) Horizontal distributions of dFe-to-N ratio (nanomolar/micromolar) at isopycnal surface 26.6 σ_θ_ in the North Pacific. (*B*) dFe vs. nitrate-plus-nitrite (N) plots, with Fe and N demand ratio by dominated diatom [the black solid line used 3 μmol Fe/mol C (42) to calculate Fe vs. N], in Okhotsk Sea shelf and the East Sakhalin. (*C*) Same as *B*, but data around the Kuril strait. (*D*) Same as *B*, but data from 155°E to the west. (*E*) Same as *B*, but data from 155°E to the East. (*F*) Same as *B*, but data around the Aleutian straits and in the Bering Sea Basin. Color in *B*–*F* indicate water density (σ_θ_). In areas *B–D*, the upper-intermediate water has high dFe concentration relative to N at a level to relax and release iron limitation. In areas *E* and *F*, the upper-intermediate water does not contain sufficient Fe for diatom growth. (*G*) Horizontal distributions of depth at isopycnal surface 26.6 σ_θ_, at which depth is outcropped by winter mixing processes in the western subarctic and the Oyashio–Kuroshio transition zone.

The intermediate water chemical properties are crucial for the productivity of the North Pacific. The nutrient circulation in the SINP coupled with the dFe in U-intermediate water derived from the Okhotsk Sea (external Fe input) (Figs. 1 *B* and *D* and 4*B*) leads to a relatively high dFe:N ratio in the intermediate waters. The dFe:N ratios are higher in the Okhotsk Sea and around the Kuril ICs in the subsurface to intermediate density ranges (Fig. 5 *A*–*C*) than that around the Aleutian ICs and in the Bering Sea basin (Fig. 5*F*), indicating that the Fe-rich water diapycnally upwells to the surface around the Kuril ICs by strong turbulent mixing (Fig. 5 *A*–*C*). The water, which has a high dFe:N ratio, spreads downstream along the Oyashio; the ratio remains high west of 155°E along the U-intermediate water pathway (Fig. 5 *A* and *D*), while the ratio decreases rapidly east of 155°E (Fig. 5 *A* and *E*), probably because of mixing with low-Fe water and scavenging during water transport. In the western subarctic and the Oyashio–Kuroshio transition zone, the upper rim of U-NPIW (isopycnal surface, 26.6 σ_θ_), which also has a relatively high dFe:N ratio (Fig. 5*A*), is able to influence the surface water because the shallower isopycnal surfaces at 26.6 σ_θ_ (∼120 m) in the western subarctic Pacific outcrop to the surface in wintertime (Fig. 5 *D* and *G*) (15, 38). The area where these waters with the high dFe:N ratio outcrop corresponds to the area where greater nutrient and biological *p*CO_*2*_ drawdown occur (4, 39). Although the Fe supply is not high enough to prevent Fe limitation (11), which causes persistent HNLC in the subarctic Pacific including around the ICs (40) and the western Bering Sea Basin, Fe supplied from the intermediate water stimulates diatom blooms in the western subarctic and the Oyashio–Kuroshio transition zone (41).

Our results clearly indicate that, in the subarctic Pacific, where high nutrients are distributed (43) at the end of global nutrient circulation (7), subpolar marginal seas and intermediate waters play pivotal roles for linking deep water to surface biogeochemistry and leading an area with among the highest nutrient concentration in the surface water (43) and the largest biological *p*CO_2_ drawdown area in the world ocean (4). We showed the processes determining the chemical properties of intermediate waters (including NPIW), and the mechanisms that determine how intermediate waters affect the supply of Fe and nutrients to the main thermocline and maintain surface productivity. The chemical properties of NPIW likely have a strong influence on biological productivity not only at high latitudes but also at low latitudes in subtropical area due to nutrient and Fe entrainment in the North Pacific (6). The subpolar marginal seas are changing under the influence of climate change, with changes such as the weakening of ventilation and intermediate water circulation with decreasing sea ice formation (44). Therefore, our findings have important implications for predicting the impact of climate change on the global nutricline, biological productivity, and the carbon cycle.

## Methods

### Field Observations.

Comprehensive observations for investigating Fe in the North Pacific were carried out from 1998 to 2018. Vertical profiles of dFe concentrations were collected in 24 cruises, which included marginal seas. All cruises that observed the dFe data are listed in *SI Appendix*, Table S1. Seawater from the surface to bottom layers was collected with acid-cleaned Teflon-coated 10- or 12-L Niskin-X bottles that were mounted on a conductivity–temperature–depth (CTD) system (SBE 9 plus) with a carousel multisampling system (SBE32) during all cruises in this study. The details of the sampling methods used for each cruise have been described elsewhere (11, 24–26, 41, 45, 46).

### dFe Measurements.

To subsample from the Niskin-X sampler during the R/V *Hakuho Maru* cruise, the samplers were transported in a clean air bubble (filled with air that had been passed through a high-efficiency particulate air filter). To subsample from the Niskin-X sampler during the R/V *Professor Multanovskiy*, *Professor Kromov* cruise, the samplers were placed in a clean tent. A 0.22-μm Millipak filter (Millipore) or a 0.2-μm Acropak filter (Pall Company) was connected to the Niskin-X spigot; then, the filtrate was collected in acid-cleaned 125-mL low-density polyethylene bottles (Nalgene Company). We confirmed that there were no significant differences between the dFe concentrations measured using the Acropak filter and the Millipak filter (*SI Appendix*, Fig. S6*B*).

Before 2006, the filtrate (<0.22 and 0.2 μm) was directly adjusted to pH 3.2 with a formic acid (10 M)–ammonium (2.4 M) buffer. After 2006, the filtrate (<0.22 and 0.2 μm) was adjusted to pH <2 by the addition of ultrapure HCl (Tamapure AA-10) and then allowed to remain at least for 24 h to 3 mo at room temperature in the onboard clean room. Each sample was then adjusted to pH 3.2 just before measurements by the addition of an ammonium solution and a formic acid (10 M)–ammonium (2.4 M) buffer. Then, dFe, defined as the leachable Fe in the filtrate at pH <2, was analyzed in the onboard or onshore laboratory using a flow-injection analysis (FIA) chemiluminescence detection system (47). All sample treatments were performed under laminar flow in the onboard or onshore clean-air laboratory. We confirmed that there were no significant differences between these two different acidified methods for open ocean sample (*SI Appendix*, Fig. S6*A*).

The quality of dFe measurements was controlled by measuring house standard seawater. Additionally, the dFe measurements and reference seawater analyses in this study after 2006 were quality-controlled using SAFe (sampling and analysis of iron) cruise (48) reference standard seawater (obtained from the University of California, Santa Cruz, for an intercomparison study). We measured a SAFe reference sample during every sample measurement run of the FIA instrument performed in the onboard and onshore laboratories in the cruise for the GEOTRACES program (*SI Appendix*, Table S1). The consensus values for Fe(III) in the SAFe reference standard seawater are 0.093 ± 0.008 nM (S) and 0.933 ± 0.023 nM (D2) (May 2013; https://www.geotraces.org/), and in GEOTRACES official cruise, for instance, we obtained values of 0.098 ± 0.010 nM (*n* = 12) (S) and 0.976 ± 0.101 nM (*n* = 10) (D2) using our method. This good agreement demonstrates that our data quality was high and that our data are comparable with the global GEOTRACES dataset. The detection limit [three times the SD of the Fe(III) concentration (0.036 nM) of purified seawater that had been passed through an 8-quinolinol resin column three times to remove Fe] was 0.020 nM. See refs. 11, 24–26, 41, 45, 46.

### Nutrient Measurements.

Nutrient (nitrate-plus-nitrite, phosphate, silicate) concentrations were also analyzed in water samples collected from the same stations. Nutrient concentrations were measured using a BRAN-LUEBBE autoanalyzer (TRACCS 800), and a BL-Tec autoanalyzer (QuAAtro). Most of the nutrient measurements in this study were quality controlled using KANSO reference material (KANSO Company). In this study, we also cited nutrient data from JAMSTEC MR04-04 cruise (31).

### Other Parameters.

Salinity and temperature were measured using a CTD sensor, and DO concentrations were measured using an oxygen sensor connected to a CTD. The DO concentration was also measured on board by the Winkler titration method, and the DO concentration obtained by the sensor was calibrated using the concentration determined by the Winkler method. The oxygen solubility was calculated and apparent oxygen utilization (AOU) was then calculated as the difference between the solubility and the measured DO concentration.

### Calculation for Percentage of Reg-PO_4_.

The percentage of reg-PO_4_ in total PO_4_ was calculated by the equation: (AOU × R_P:DO_)/observed PO_4_ × 100. In this equation, R_P:DO_ is a regenerated mole ratio for phosphate to oxygen; we employed a R_P:DO_ of 170 from ref. 49.

### Estimating Vertical Fluxes of dFe and Nitrate.

The material flux was estimated at the Kuil and the Aleutian ICs. We employed a simple calculation to estimate the vertical flux of dFe and N from the subsurface to the surface at the ICs using the following equations:

$$\mathrm{dFe}\;\mathrm{Flux}\;=K_{\rho }\times \left(\mathrm{dFe}/\mathrm{dz}\right),\;\;N\;\mathrm{Flux}\;=K_{\rho }\times \left(\mathrm{dN}/\mathrm{dz}\right).$$

Our measured dFe and N vertical profiles at the ICs strait were already influenced by the strong mixing, and the gradients (dFe/dz and dN/dz) in the profiles from surface to subsurface were disrupted. Thus, the gradients in the profiles at the IC strait were not suitable for estimating the material flux from intermediate water to surface water. To evaluate the flux from the intermediate water to the surface water at the IC straits, we used the vertical profile of dFe and N obtained around the straits (locations are blue dots in See *SI Appendix*, Fig. S7), which we used to approximate the profiles before the water was influenced by the mixing process. The surface to subsurface gradients of dFe (dFe/dz) and N (dN/dz) were evaluated at all stations located around the straits (blue dots for the ICs and yellow dots for the subarctic Pacific). To estimate fluxes, we combined the gradients with the measured snapshot of vertical diffusivity *K*_*ρ*_ (=0.2*εN*^−2^, where *ε* is turbulent kinetic energy dissipation rate in watts per kilogram and *N*^2^ = −*gρ*_*z*_*/ρ*, where *N*^2^ is squared buoyancy frequency, and where *g* and *ρ* are the gravitational acceleration and reference potential density, respectively) for depths of 100 to 500 m. The *K*_*ρ*_ was measured by using a free-fall vertical microstructure profiler (VMP2000; Rockland Scientific International Company) (32–34) on the cruises Kh06, Kh07, and KH-09-4 for the IC waters and on the cruise KH-08-2 for the open waters in the western subarctic Pacific (*SI Appendix*, Table S2). The *K*_*ρ*_ was also measured by using CTD-attached fast-response thermistors (AFPO7; Rockland Scientific International Company) (50, 51) on the KH-17-3 cruise for open water in the subarctic Pacific (*SI Appendix*, Table S2). Comparison study between these two measurement methods have been conducted at 100 stations in several cruises (51), including KH-09-4 (*SI Appendix*, Table S2) (51). Turbulence intensity estimated from CTD-fast-response thermistors was compared to those by free-fall microstructure profilers, conducted at the same location within 2 h, and the result was reported in Goto et al. (51), where *ε* is valid for 10^−10^ < *ε* <10^−8^ W/kg after response correction (50) and data screening (51), and it has been confirmed that *ε* from both measurement methods are comparable and within a factor of 3 (51).

### Estimated Budget of Nitrate-plus-Nitrite (N) Among the Surface, Intermediate, and Deep Waters (*SI Appendix*, Figs. S4 and S8).

The annual transport of N from the SINP to the surface was calculated by the geometric mean of uplifted N fluxes at the Kuril and Aleutian ICs accounting for the approximate area where mixing occurs [the Kuril ICs, 1.12469E + 11 m^2^, and Aleutian ICs, 2.14087E + 11 m^2^; the IC areas were defined to cover where the depth-integrated tidal energy dissipation rate estimated from a global barotropic tide model (52) were higher than 5.0 × 10^−2^ W/m^2^ along the ICs] (*SI Appendix*, Fig. S8). The uplifted N flux in the open ocean in the subarctic Pacific was calculated by the geometric mean fluxes in the subarctic Pacific, accounting for the area of the northern WSG (4.92255E + 12 m^2^) and Alaskan Gyre (3.02873E + 12 m^2^). Exported N from the surface through the winter mixed layer depth was calculated by using number of 1.49 to ∼2.3 mol-C⋅m^−2^⋅y^−1^, which was previously reported in the WSG (35, 36) and the Alaskan Gyre (36), accounting for the area of the northern WSG and Alaskan Gyre (*SI Appendix*, Fig. S8). N pooled in the SINP was calculated with an average N concentrations (42 ± 2.3 μmol/kg) in the intermediate water range between 26.8 and 27.6 at the stations, the thickness of the intermediate water (1237 ± 137 m), in the whole subarctic Pacific and the western Bering sea, and the area of the northern WSG and Alaskan Gyre (*SI Appendix*, Fig. S8).

### Ocean Data View Parameters.

Ocean Data View (ODV) (https://odv.awi.de/) (53) was used to calculate and produce plots for the basin-scale isopycnal surface distribution and vertical section profiles of each parameter in Figs. 1–3 and 5 and *SI Appendix*, Figs. S1, S2, S5, and S7.

### Data Availability.

The data that support the findings of this study are available at https://eprints.lib.hokudai.ac.jp/dspace/handle/2115/77482.

## Supplementary Material

Supplementary File
