# Supplementary material for: Subpolar marginal seas fuel the North Pacific through the intermediate water at the termination of the global ocean circulation
Source: Proc Natl Acad Sci U S A. 2020 May 27;117(23):12665–73. doi: 10.1073/pnas.2000658117 (PMC7293607; doi:10.1073/pnas.2000658117)
Supplement: Supplementary File [file pnas.2000658117.sapp.pdf]

**Supporting Information for**

# Sub-polar marginal seas fuel the North Pacific through the intermediate water at the termination of the global ocean circulation

Jun Nishioka[a,b]*, Hajime Obata[c], Hiroshi Ogawa[c], Kazuya Ono[a], Youhei Yamashita[d], Keun Jong Lee[c], Shigenobu Takeda[e] and Ichiro Yasuda[c]

[a]*Pan-Okhotsk Research Center, Institute of Low Temperature Science, Hokkaido University, Sapporo, Japan*
[b]*Arctic Research Center, Hokkaido University*
[c]*Atmosphere and Ocean Research Institute, The University of Tokyo, Kashiwa, Japan*
[d]*Faculty of Environmental and Earth Science, Hokkaido University, Sapporo, Japan*
[e]*Graduate School of Fisheries and Environmental Sciences, Nagasaki University, Nagasaki Japan*

*Corresponding author
Jun Nishioka: +81-11-706-7655, nishioka@lowtem.hokudai.ac.jp
ORC-ID: 0000-0003-1723-9344

## Supporting Information

**Supporting information Table 1**

List of cruises collected data of this study

| Year | Month | Ship | Cruise ID | area | Filter type | Acidify before measurement | reference | |
|---|---|---|---|---|---|---|---|---|
| 1998 | Aug-Sep | R/V J.P. Tully | 9829 | Gulf of Alaska LineP | Millipak | pH 3.2 | ref SI (1) | Nishioka et al., 2001 |
| 2000 | May-June | R/V Mirai | MR00-K03 | Western subarctic Pacific, Kuril island, Okhotsk sea | Millipak | pH 3.2 | ref SI (2), (3) | Nishioka et al., 2007, Nishioka et al., 2003 |
| 2003 | Jan | R/V Hokko-maru | HK0301 | Oyashio area | Millipak | pH 3.2 | ref SI (4) | Nishioka et al., 2011 |
| 2003 | Feb | T/V Oshoro-Maru | OS0302 | Oyashio area | Millipak | pH 3.2 | ref SI (4) | Nishioka et al., 2011 |
| 2003 | Mar | T/V Oshoro-Maru | OS0302 | Oyashio area | Millipak | pH 3.2 | ref SI (4) | Nishioka et al., 2011 |
| 2003 | April | R/V Wakataka-Maru | WK0304 | Oyashio, western North Pacific | Millipak | pH 3.2 | ref SI (2), (4) | Nishioka et al., 2007, Nishioka et al., 2011 |
| 2003 | May | R/V Wakataka-Maru | WK0305 | Oyashio, western North Pacific | Millipak | pH 3.2 | ref SI (2), (4) | Nishioka et al., 2007, Nishioka et al., 2011 |
| 2003 | Sep-Oct | R/V Hakuho-Maru | KH-03-2 | Western North Pacific, 155 E line | Millipak | pH 3.2 | ref SI (2) | Nishioka et al., 2007 |
| 2005 | Jan | R/V Hokko-maru | HK0501 | Oyashio area | Millipak | pH 3.2 | ref SI (4) | Nishioka et al., 2011 |
| 2005 | May | R/V Hokko-maru | HK0505 | Oyashio area | Millipak | pH 3.2 | ref SI (4) | Nishioka et al., 2011 |
| 2005 | Dec | R/V Hokko-maru | HK0512 | Oyashio area | Millipak | pH 3.2 | ref SI (4) | Nishioka et al., 2011 |
| 2006 | Jan | R/V Hokko-maru | HK0601 | Oyashio area | Millipak | pH 3.2 | ref SI (4) | Nishioka et al., 2011 |
| 2006 | July-Aug | R/V Pro. Khromov | Kh06 | Kuril Ics, Okhotsk sea | Millipak | pH < 2 | ref SI (5), (6) | Nishioka et al., 2013 Nishioka et al., 2014 |
| 2007 | July-Aug | R/V Pro. Khromov | Kh07 | Kuril Ics, Okhotsk sea | Millipak | pH < 2 | ref SI (6) | Nishioka et al., 2014 |
| 2008 | Aug-Sep | R/V Hakuho-Maru | KH-08-2 | Western North Pacific, 155 E line | Millipak | pH < 2 | ref SI (5) | Nishioka et al., 2013 |
| 2009 | Aug-Sep | R/V Hakuho-Maru | KH-09-4 | Aleutian Islands－Bering sea | Millipak | pH < 2 | This study | This study |
| 2010 | June-July | R/V Pro. Khromov | Kh10 | Kuril Ics, Okhotsk sea | Millipak | pH < 2 | This study | This study |
| 2012 | July-Aug | R/V Hakuho-Maru | KH-12-3 | Western North Pacific, 160 E line | Acropac | pH < 2 | ref SI (7) | Yamashita et al., 2020 |
| 2012 | July-Aug | R/V Hakuho-Maru | KH-12-4 | subarctic North Pacific, GP02 GEOTRACES | Acropac | pH < 2 | ref SI (8) | Nishioka and Obata, 2017 |
| 2013 | July-Aug | T/V Oshoro-Maru | OS13 | subarctic North Pacific, Bering Sea, Surface survey | Millipak | pH < 2 | This study | This study |
| 2014 | May-June | R/V Pro. Multanovskiy | Mu14 | East Kamchatska current | Acropac | pH < 2 | This study, ref SI (7), (9) | This study※ |
| 2015 | Nov-Dec | R/V Hakuho-Maru | KH-15-4 | Kuroshio area | Acropac | pH < 2 | This study | This study |
| 2017 | June-Aug | R/V Hakuho-Maru | KH-17-3 | subarctic North Pacific, GP02 GEOTRACES | Acropac | pH < 2 | This study | This study |
| 2018 | July-Sep | R/V Pro. Multanovskiy | Mu18 | Western Bering Sea | Acropac | pH < 2 | This study | This study |

※ Part of this cruise Fe and nutrient data were used in ref SI (7) and (9).

※※ Nutrient data in this studies data set include the data referred from JAMSTEC Mirai, MR04-04 cruise, http://www.godac.jamstec.go.jp/darwin/cruise/mirai/mr04-04/j

**Supporting information Table 2**

Estimated N & dFe fluxes in the subarctic Pacific ocean

| Cruise | Station for estimate Nitrate Flux | Latitude | Lognitude | K (100-500m) (m2/s) | dN/dz | Station wich is used for dN/dZ caluculation | N flux | ddFe/dz | Station wich is used for ddFe/dZ caluculation | dFe flux |
|---|---|---|---|---|---|---|---|---|---|---|
| | | | | | (mmol/m4) | | (mmol/m2/day) | (umol/m4) | | (umol/m2/day) |
| Kh06 | BF | 46.43333 | 151.1167 | 1.00.E-03 | 0.036 | A6 | 3.13E+00 | 0.0046 | A6 | 3.97E-01 |
| Kh06 | Urup-E | 46.36667 | 150.65 | 1.10.E-03 | 0.036 | A6 | 3.44E+00 | 0.0046 | A6 | 4.37E-01 |
| Kh06 | Urup-W | 45.16667 | 149.4 | 3.63.E-05 | 0.036 | A6 | 1.14E-01 | 0.0046 | A6 | 1.44E-02 |
| Kh06 | Bussol-9 | 46.56667 | 151.5167 | 3.44.E-04 | 0.036 | A6 | 1.08E+00 | 0.0046 | A6 | 1.37E-01 |
| Kh06 | Bussol-13 | 46.71667 | 151.7167 | 4.99.E-01 | 0.036 | A6 | 1.56E+03 | 0.0046 | A6 | 1.98E+02 |
| Kh07 | KC2 | 48.2783 | 153.545 | 1.79.E-04 | 0.050 | B1 | 7.73E-01 | 0.0033 | B1 | 5.17E-02 |
| Kh07 | BF1 | 46.4433 | 151.1233 | 2.55.E-03 | 0.050 | B1 | 1.10E+01 | 0.0033 | B1 | 7.36E-01 |
| Kh07 | BB3 | 46.5833 | 151.5333 | 6.53.E-04 | 0.050 | B1 | 2.82E+00 | 0.0033 | B1 | 1.88E-01 |
| Kh10 | UE | 46.21667 | 150.8 | 6.34.E-03 | 0.086 | A6 | 4.71E+01 | 0.0042 | A6 | 2.28E+00 |
| Kh10 | BF1 | 46.43333 | 151.1167 | 9.96.E-04 | 0.086 | A6 | 7.40E+00 | 0.0042 | A6 | 3.58E-01 |
| Kh10 | BW1 | 46.58333 | 151.1167 | 9.41.E-05 | 0.086 | A6 | 7.00E-01 | 0.0042 | A6 | 3.38E-02 |
| Kh10 | BNK1 | 47.45 | 154 | 5.71.E-04 | 0.122 | A1 | 6.02E+00 | 0.0033 | A1 | 1.63E-01 |
| KH-09-4 | Near pass | 53.3335 | 170.1988 | 3.29.E-06 | 0.111 | St.6 | 3.15E-02 | 0.0024 | St.6 | 6.71E-04 |
| KH-09-4 | Buldir W pass | 52.373 | 175.2673 | 7.70.E-04 | 0.048 | St.21 | 3.18E+00 | 0.0016 | St.21 | 1.06E-01 |
| KH-09-4 | Amchitka strait | 51.50933 | 180.0498 | 3.85.E-04 | 0.048 | St.21 | 1.59E+00 | 0.0016 | St.21 | 5.32E-02 |
| KH-09-4 | Amukta strait | 52.402 | 188.1077 | 2.86.E-03 | 0.051 | St.51 | 1.26E+01 | 0.0020 | St.51 | 4.84E-01 |
| KH-08-2 | KNOT | 44 | 155 | 1.60.E-06 | 0.240 | KNOT | 3.32E-02 | 0.0034 | KNOT | 4.68E-04 |
| KH-17-3 | CL2 | 47 | 160 | 2.33.E-05 | 0.086 | CL2 | 1.72E-01 | 0.0025 | CL2 | 5.03E-03 |
| KH-17-3 | CL4 | 47 | 175 | 1.15.E-04 | 0.069 | CL4 | 6.82E-01 | 0.0017 | CL4 | 1.69E-02 |
| KH-17-3 | CL6 | 47 | 195 | 2.79.E-05 | 0.085 | CL6 | 2.03E-01 | 0.0013 | CL6 | 3.13E-03 |
| KH-17-3 | CL8 | 47 | 205 | 1.02.E-06 | 0.061 | CL8 | 5.41E-03 | 0.0008 | CL8 | 6.64E-05 |
| KH-17-3 | CL10 | 47 | 210 | 1.04.E-05 | 0.066 | CL10 | 5.90E-02 | 0.0006 | CL10 | 5.68E-04 |
| KH-17-3 | CL16 | 50 | 215 | 8.57.E-05 | 0.085 | CL16 | 6.26E-01 | 0.0008 | CL16 | 5.85E-03 |

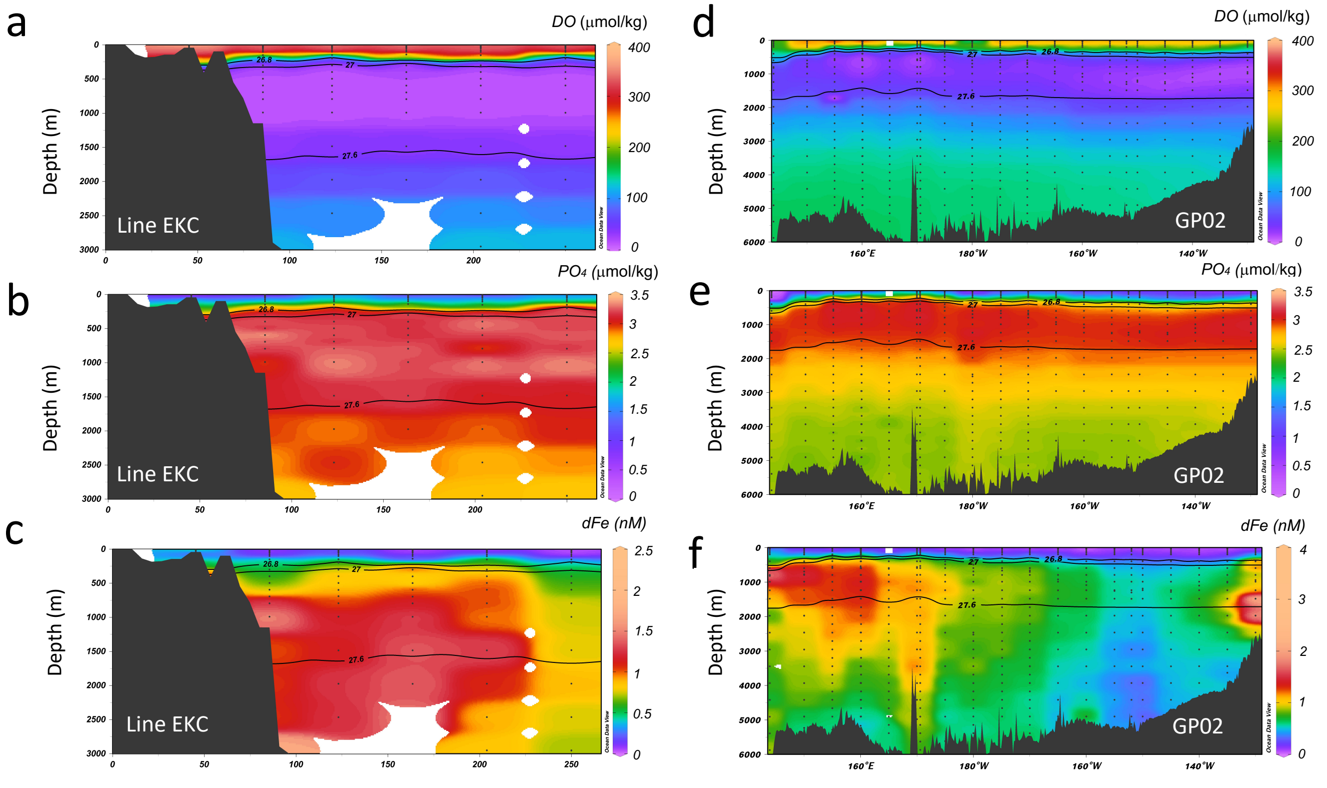


**Supporting information Figure 1**

a) Vertical section profile of dissolved oxygen along line EKC in Figure 2b, b) same as a) but for phosphate, c) same as a) but for dissolved Fe. d) same as a) but along GP02, e) same as a) but for phosphate along GP02, f) same as a) but for dissolved Fe along GP02. Black solid line indicate isopycnal surface 26.8, 27.0, 27.6 $\sigma_\theta$.

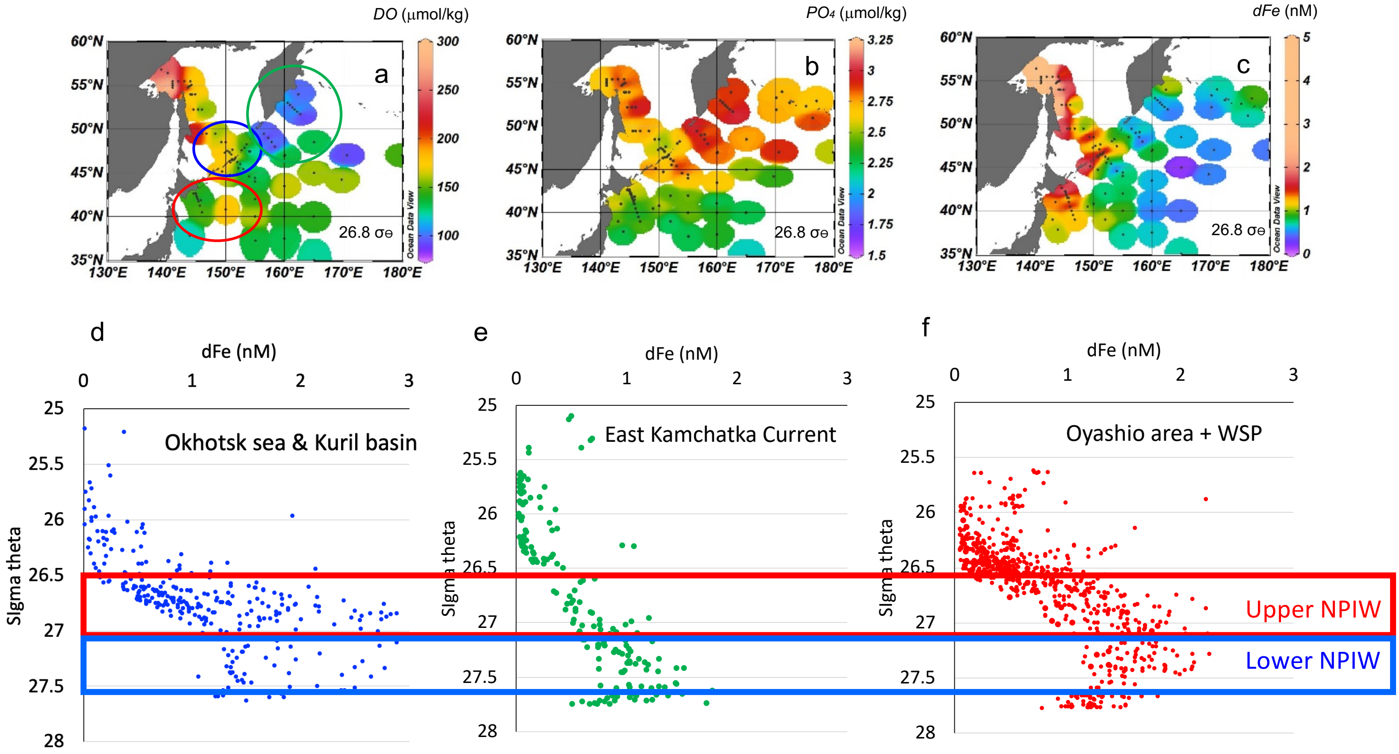


**Supporting information Figure 2**

a) Horizontal distribution of dissolved oxygen concentration (μmol/kg) at isopycnal surface 26.8 $\sigma_\Theta$, b) same as a) but for phosphate concentration (μmol/kg) , c) same as a) but dissolved Fe (nM), d) vertical profile of dissolved Fe vs density around the Kuril Basin and Okhotsk Sea (blue circle in a)), e) same as d) but along the East Kamchatska Current (green circle in a)), f) same as d) but around the Oyashio+WSP area (red circle in a))

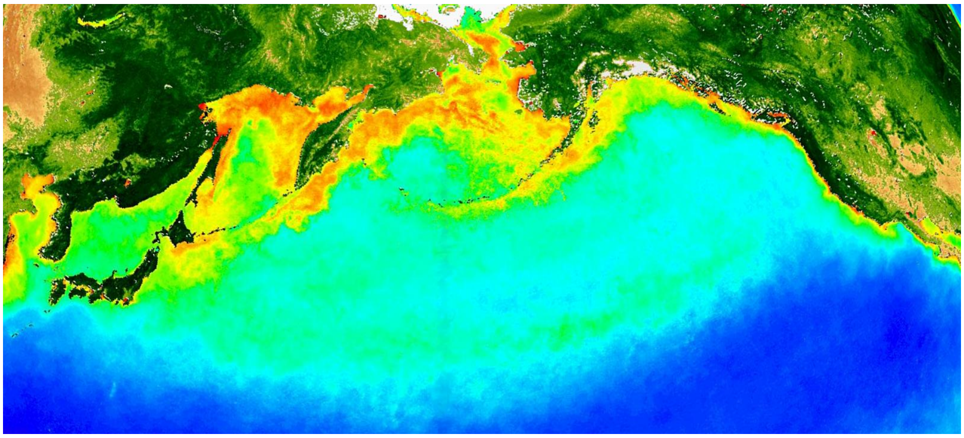

**Supporting information Figure 3**
Satellite chlorophyll image in the North Pacific Ocean provided by Seawifs project (https://oceancolor.gsfc.nasa.gov/SeaWiFS/)

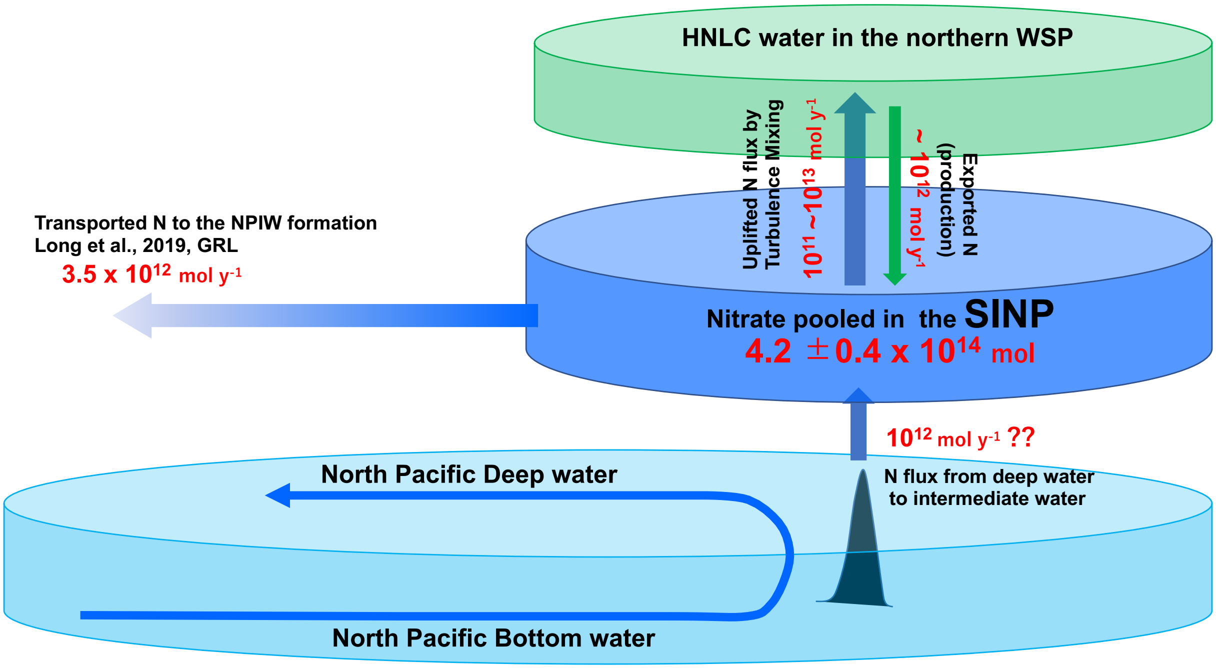


**Supporting information Figure 4**

Estimated budget of Nitrate+Nitrite (N) among the surface water, the SINP water and deep water. Account for the area of turbulent mixing occur, the estimated uplifted annual N fluxes from the SINP to surface around the Aleutian, the Kuril ICs is $10^{11}$~$10^{13}$ mol $y^{-1}$ (geometric mean;$10^{12}$ mol $y^{-1}$). The estimated flux of uplifted N only by turbulent mixing in the open ocean in the subarctic Pacific is ~ $10^{11}$ mol $y^{-1}$. Exported N production from surface to below the winter mixed layer depth in the northern subarctic gyre is ~$10^{12}$ mol $y^{-1}$ (calculated with number cited by (ref SI, 10, 11)). N pooled in the SINP is 4.2 ±0.4 × $10^{14}$ mol in the whole subarctic Pacific (calculated by this study's data set). Transported N to the NPIW formation is referred by (ref SI, 12). The geometric mean of total uplifted annual N flux estimated in this study (~ $10^{12}$ mol $y^{-1}$, See Fig. S8), however, might be underestimated or there is another missing upward flux of N (or exported N might be overestimated) because uplifted N flux must be greater than exported N for maintaining high nutrient surface water in the subarctic Pacific.

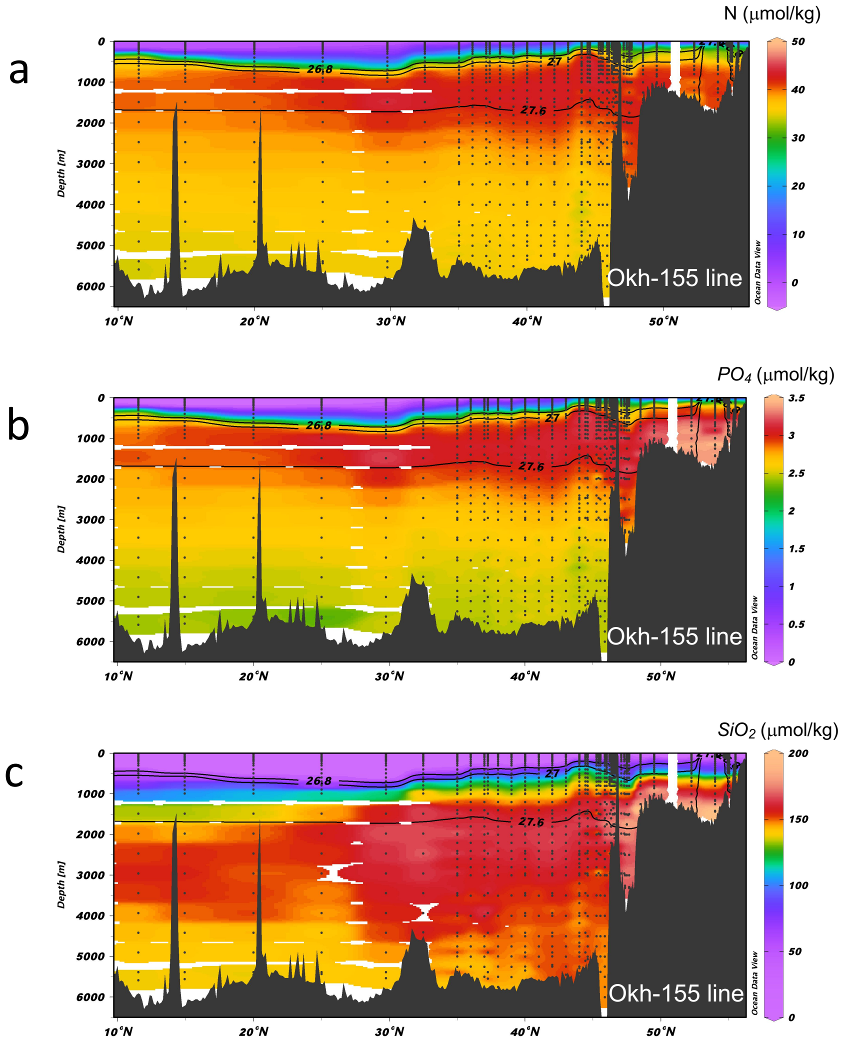


**Supporting information Figure 5**

a) Vertical section profile of nitrate+nitrite along Okh-155 line in Figure 2b, b) same as a) but for phosphate, c) same as a) but for silicate.

a

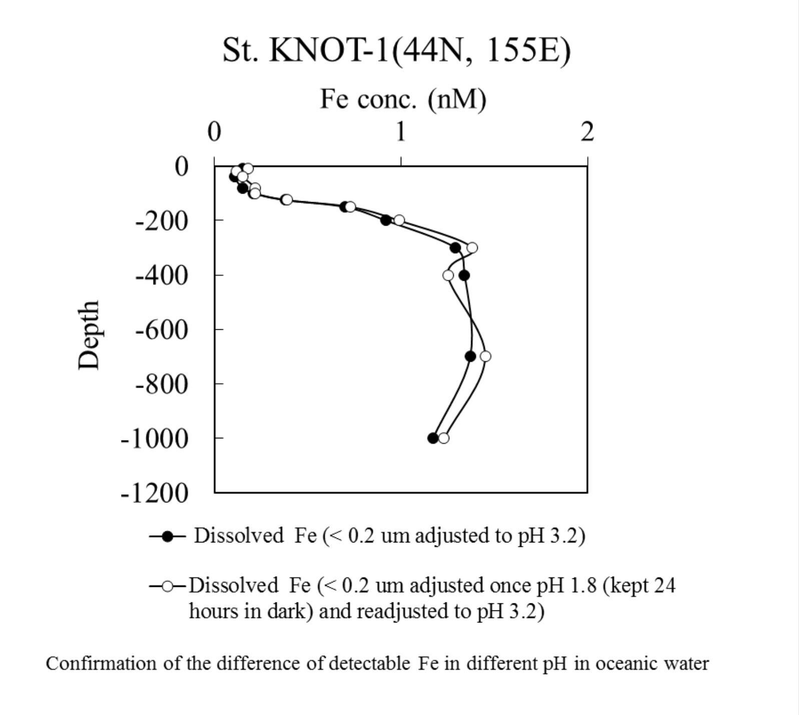


b

| filter check | | Fe (nM) | STDEV |
|---|---|---|---|
| 1000m | Acropac NO pressure | 0.41 | 0.008 |
| | Acropac pressure | 0.44 | 0.001 |
| | | | |
| | Millipac NO pressure | 0.46 | 0.005 |
| | Millipac pressure | 0.46 | 0.003 |

**Supporting information Figure 6**

a) Results of comparison of measurement acidification between sample which directly adjust pH to 3.2 and once adjust pH below 2.0. There are no significant differences between in the open ocean water at 44˚ N, 155 ˚ E profile, but we observed significant difference in coastal and marginal sea waters. b) Comparison of filters which used in this study. There are no significant differences between dFe concentrations measured using the Acropak filter and the Millipak filter.

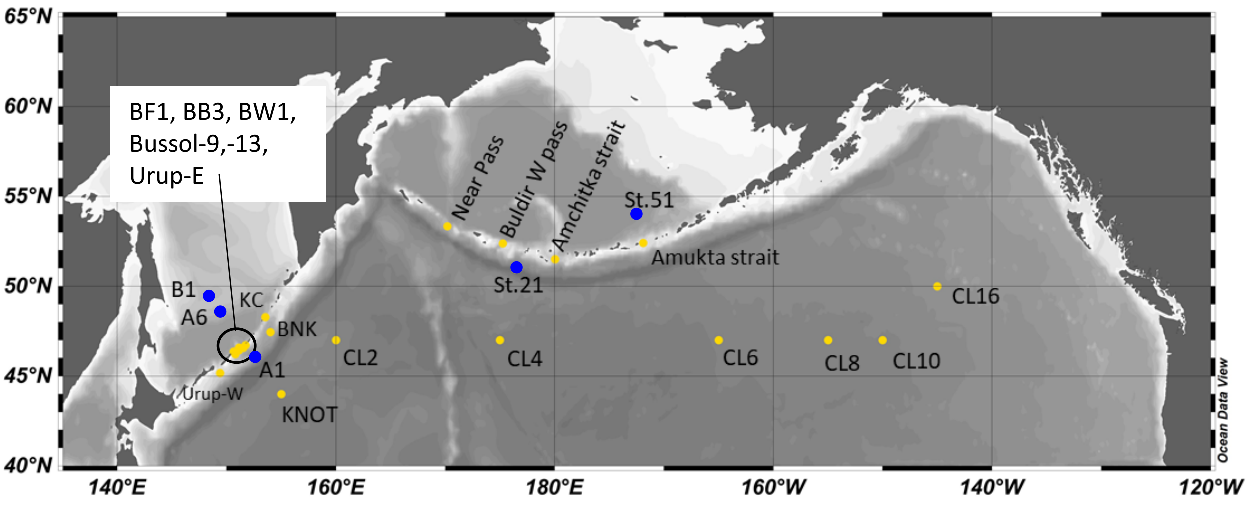


**Supporting information Figure 7**

Location of estimate for nitrate+nitrite (N) fluxes. Yellow dots are indicating location where turbulent diffusivity measured and the fluxes were estimated. Blue dots indicate location where vertical distributions obtained gradients of Fe and N for estimating fluxes at the Kuril and the Aleutian IC areas (See Supplementary Table 2).

WSP: 4,922,553 $km^2$

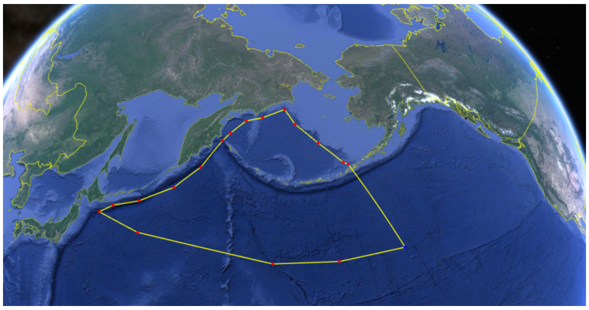

AG: 3,028,730 $km^2$

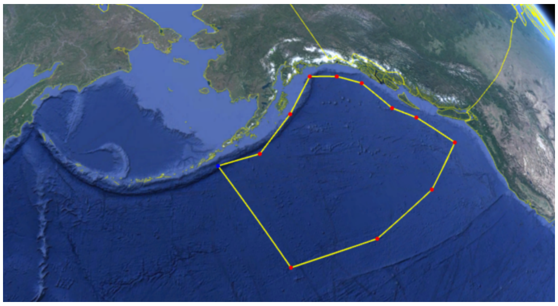

Aleutian ICs: 214,087 $km^2$

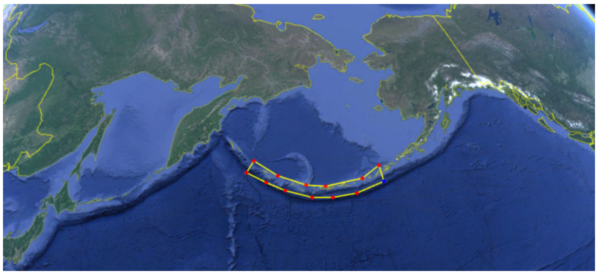

Kuril ICs: 112,469 $km^2$

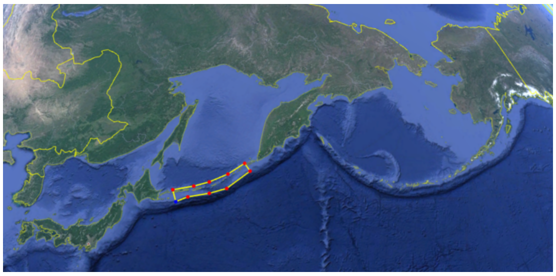

a) Nitrate pooled in the SINP
= average conc (42.6 ± 2.3 $mmol/m^3$; from this study) x average depth thickness 26.8-27.6 (1237 ± 37; from this study) x SINP area (WSP+AG) = 4.2 ± 0.4 E+14 mol

---

b) Upward Flux from the SINP to surface

Upward Nitrate Flux around Aleutian ICs (1)
= Aleutian ICs N flux (geometric mean) = (1.2 E+00 $mmol/m^2/day$ ; from this study) x Aleutian ICs area
= 0.9 E+11 mol/year

Upward Nitrate Flux around Kuril ICs (2)
= Kuril ICs N flux (geometric mean) = (4.5 E+00 $mmol/m^2/day$ ; from this study) x Kuril ICs area
= 1.9 E+11 mol/year

Upward Nitrate Flux in the WSP and AG (3)
= Open Ocean N flux (geometric mean) = (1.1 E-01 $mmol/m^2/day$ ; from this study) x (WSP+AG) area
= 3.1 E+11 mol/year

Total uplifted N flux = (1) + (2) + (3) = 0.6 E+12 mol/year

---

c) Exported N from surface to deep

2.3 ± 0.3 mol-C/$m^2$/year (Palevsky et al., 2016) and 1.49 ± 0.42 mol-C/$m^2$/year (Wakita et al 2016) and Redfield ratio (106:16 for C:N) were used for estimating export N from surface area of WSP+AG. The values are in the range of 1.79 ~2.76 E+12 mol-N/year.

**Supporting information Figure 8**
Areas which are used for estimation of of Nitrate+Nitrite (N) budget among the surface water, the SINP water in the Supporting information Figure 4. The IC areas are defined to cover where the depth-integrated tidal energy dissipation rate estimated from a global barotropic tide model (ref SI, 13) were higher than $5.0 \times 10^{-2}$ $W/m^2$ along the ICs. These areas are estimated by Google Earth tool. The equation for the estimation of a) Nitrate pooled in the SINP, b) Upward Flux from the SINP to surface, c) Exported N from surface to deep are also indicated.

References


1. J. Nishioka, S. Takeda, C. S., W. K. Wong, Johnson Size-fractionated iron concentrations in the northeast Pacific Ocean: distribution of soluble and small colloidal iron. Mar. Chem. 74, 157-179, doi.org/10.1016/S0304-4203(01)00013-5 (2001)
2. J. Nishioka et al., Iron supply to the western subarctic Pacific: Importance of iron export from the Sea of Okhotsk. J. Geophys. Res. 112, C10012 https://doi.org/10.1029/2006JC004055 (2007).
3. J. Nishioka et al., Size-fractionated iron distributions and iron-limitation processes in the subarctic NW Pacific, Geophys. Res. Lett. 30(14), doi:10.1029/2002GL016853 (2003).
4. J. Nishioka, T. Ono, H. Saito, K. Sakaoka, T. Yoshimura, Oceanic iron supply mechanisms which support the spring diatom bloom in the Oyashio region, western subarctic Pacific. J. Geophys. Res. 112, C10012, doi: 10.1029/2010JC006321 (2011).
5. J. Nishioka et al., Intensive mixing along an island chain controls oceanic biogeochemical cycles. Global Biogeochem. Cycle 27, 920–929 (2013).
6. J. Nishioka et al., Quantitative evaluation of iron transport processes in the Sea of Okhotsk. Prog. Oceanogr. 126, 180-193 (2014).
7. Y. Yamashita, J. Nishioka, H. Obata, H. Ogawa, Shelf humic substances as carriers for basin-scale iron transport in the North Pacific, Scientific Reports, 10, 4505, doi.org/10.1038/s41598-020-61375-7 (2020).
8. J. Nishioka, H. Obata, Dissolved iron distribution in the western and central subarctic Pacific: HNLC water formation and biogeochemical processes. Limnol. Oceanogr. 62 (5), 2004-2022 (2017).
9. K. Yoshida, S. Nakamura1, J. Nishioka, S. B. Hooker, K. Suzuki, Community composition and photosynthetic physiology of phytoplankton in the western subarctic Pacific near the Kuril Islands with special reference to iron availability. accepted in J. Geophys. Res. Biogeoscience, doi:10.1029/2019JG005525 (2020).
10. H. I. Palevsky, P. D. Quay, D. E. Lockwood, D. P. Nicholson, The annual cycle of gross primary production, net community production, and export efficiency across the North Pacific Ocean. Global Biogeochem. Cycle, 361-380, doi:10.1002/2015GB005318 (2016).
11. M. Wakita et al., Biological organic carbon export estimated from the annual carbon budget observed in the surface waters of the western subarctic and subtropical North Pacific Ocean from 2004 to 2013. J. Oceanogr. 72, 665- 685, 10.1007/s10872-016-0379-8 (2016).
12. Y. Long, X-H. Zhou, X. Guo, The Oyashio Nutrient Stream and its Nutrient Transport to the Mixed Water Region. Geophys. Res. Lett. 46, 1513–1520, doi.org/10.1029/2018GL081497 (2019).
13. Y. Tanaka, I. Yasuda, H. Hasumi, H. Tatebe, and S. Osafune, 2012: Effects of the 18.6-year modulation of tidal mixing on the North Pacific bidecadal climate variability in a coupled climate model, Journal of Climate, 25, 7625-7642 (2012).
